# MYD88 Is a Potential Prognostic Gene and Immune Signature of Tumor Microenvironment for Gliomas

**DOI:** 10.3389/fonc.2021.654388

**Published:** 2021-04-07

**Authors:** Qinglong Guo, Xing Xiao, Jinsen Zhang

**Affiliations:** ^1^ Department of Neurosurgery, Huashan Hospital, Shanghai Medical College, Fudan University, Shanghai, China; ^2^ Neurosurgery Department of Huashan Hospital, Neurosurgical Institute of Fudan University, Shanghai, China; ^3^ Neurosurgery Department of Huashan Hospital, Shanghai Clinical Medical Center of Neurosurgery, Shanghai, China; ^4^ Shanghai Key Laboratory of Brain Function and Restoration and Neural Regeneration, Shanghai, China

**Keywords:** tumor microenvironment, tumor-infiltrating immune cell, MYD88, macrophage, glioma

## Abstract

**Purpose:**

To explore the profiles of immune and stromal components of the tumor microenvironment (TME) and their related key genes in gliomas.

**Methods:**

We applied bioinformatic techniques to identify the core gene that participated in the regulation of the TME of the gliomas. And immunohistochemistry staining was used to calculate the gene expressions in clinical cases.

**Results:**

The CIBERSORT and ESTIMATE were used to figure out the composition of TME in 698 glioma cases from The Cancer Genome Atlas (TCGA) database. Differential expression analysis identified 2103 genes between the high and the low-score group. Then the Gene Ontology (GO), Kyoto Encyclopedia of Genes and Genomes (KEGG) enrichment analysis, univariate Cox regression analysis, and protein–protein interaction (PPI) network construction were conducted based on these genes. MYD88 was identified as the key gene by the combination univariate Cox and PPI analysis. Furthermore, MYD88 expression was significantly associated with the overall survival and WHO grade of glioma patients. The genes in the high-expression MYD88 group were mainly in immune-related pathways in the Gene Set Enrichment Analysis (GSEA). We found that macrophage M2 accounted for the largest portion with an average of 27.6% in the glioma TIICs and was associated with high expression of MYD88. The results were verified in CGGA database and clinical cases in our hospital. Furthermore, we also found the MYD88 expression was higher in IDH1 wild types. The methylation rate was lower in high grade gliomas.

**Conclusion:**

MYD88 had predictive prognostic value in glioma patients by influencing TIICs dysregulation especially the M2-type macrophages.

## Introduction

Gliomas are the most common primary malignant neoplasms of the central nervous system with an incidence of five to six cases per 100,000 persons per year (1). Standard treatment of gliomas includes surgical resection, radiotherapy and chemotherapy (2, 3). The World Health Organization classified the gliomas as grade I-IV based on clinical, genetic, and histopathological criteria (4). In spite of the tremendous progress in the genetic and epigenetic landscapes of glioma, there are still no substantial survival benefits (5, 6).

Recently, increasing evidences have revealed the importance and complexity of the tumor microenvironment (TME) in tumor progression. The TME is made up of the tumor surrounding tissues, including the immune cells, stromal cells, and the extracellular matrix (7–9). Tumor cells can affect the TME by releasing molecular signals, enhancing angiogenesis, and inducing immune suppression, while the immune cells in the TME can influence the growth and evasion of tumor cells (7, 10). Tumor immunotherapies, such as immune checkpoint inhibitors, Chimeric Antigen Receptor T-Cell Immunotherapy (CAR-T), vaccine, and oncolytic virus have been introduced and achieved benefits in many cancers (11, 12). However, several large phase 3 clinical trials on PD-1 inhibitors in the treatment of GBM patients have failed to achieve survival benefits (13, 14). The comprehensive understanding of underlying molecular mechanisms of the TME could help develop new treatment strategies to improve the efficacy of immunotherapies (15). Xiangyang Deng et al. conducted bioinformatics analysis based on and identified a list of prognostic immune‐related genes (IRGs) and provided a perspective to explore the immune infiltration pattern in lower grade gliomas (WHO grade II and III) (16). In another recent study, a novel immune prognostic signature was introduced as a promising biomarker for GBM risk stratification, prognostic assessment and immunophenotypic classification (17). Low-grade gliomas (WHO grade II) have a uniform rate of recurrence and increase in grade over time. Therefore, we incorporated gliomas ranging from WHO II to WHO IV using The Cancer Genome Atlas (TCGA) database (https://portal.gdc.cancer.gov/) to figure out the TME characteristics and validated in Chinese Glioma Genome Atlas (CGGA) database (http://www.cgga.org.cn/) and clinical cases.

Myeloid differentiation primary response protein 88 (MYD88) is mainly located in the plasma and acts as an key adaptor protein in the downstream of toll-like receptor (TLR) and interleukin-1 receptor (IL-1R) signaling pathways. TLRs are the superfamily of pattern recognition receptors that activate and mediate innate and adaptive immunity (18, 19). They participate in the tumor-related immunity responses contributing to the development and progression of tumors (19–21). Recent studies have shown that TLRs could reverse tumor differentiation (22) and transform microglia into a glioma supportive phenotype in gliomas (19). Macrophages are mainly subdivided into two M1 and M2-phenotypes, which have pro-inflammatory and anti-inflammatory correspondingly. Characterizing as M2-like macrophages, tumor-associated macrophages (TAMs) occupied a large portion of the TME in gliomas and are associated with poor prognosis (23). The mutual interaction between the transformation of M2 macrophage cells and glioma cells contributed to the rapid progression of gliomas (24). The function and prognostic value of MYD88 and its related TLRs/IL-1R pathway in TME have not been fully explored in gliomas.

In this study, the Estimation of STromal and Immune cells in MAlignant Tumor tissues using Expression data (ESTIMATE) (25, 26) and Cell-type identification by estimating relative subsets of RNA transcripts (CIBERSORT) (27) were used to compute the tumor-infiltrating immune cell (TIIC) proportion and the ratio of immune and stromal components of 698 glioma samples including 529 LGG samples and 169 GBM samples from TCGA database. Then we conducted the Gene Ontology (GO) and Kyoto Encyclopedia of Genes and Genomes (KEGG) enrichment analysis, PPI analysis, univariate Cox regression analysis, and correlation analysis of TIICs and gene expression. Finally, myeloid differentiation primary response 88 (MYD88) was identified as the potential prognostic gene and immune signature of glioma TME and was significantly associated with the higher percentage of M2 macrophages. Then we validated the MYD88 expression both in the CGGA and in Thirty-one glioma patients and found similar results. Moreover, the MYD88 expression was also related with IDH1 mutation status and methylation.

## Materials and Methods

### Data and Sample Collection

Transcriptome RNA-seq data of 703 cases (including 529 LGG samples, 169 GBM samples and 5 normal samples) and clinical data were downloaded from TCGA database in September 2020. Thirty-one tissues from glioma patients were collected from the Department of Neurosurgery, Huashan Hospital, Fudan University. All patients signed an informed consent form (KY2015-256), which was approved by the Clinical Research Ethics Committee of the Huashan Hospital.

### Immunohistochemistry (IHC)

The clinical samples were fixed in 10% neutral buffered formalin for 24 h. Paraffin was used for tissue embedding. Then tissue slides were well prepared and deparaffinized using dimethylbenzene, anhydrous ethanol, 85% ethanol, 75% ethanol, and distilled water orderly. The container with ethylene diamine tetraacetic acid (EDTA) antigen repair buffer (PH 9.0, Servicebio, G1203) for MYD88 or citric acid tissue antigen repair solution (PH 6.0, Servicebio, G1202) for CD68 and CD163 in the microwave oven was used correspondingly to repair the antigen of the slides using median fire to boiling for 8 min, keeping warm for 8 min and median-low fire for 7 min consecutively. Peroxidase was blocked using the 3% H_2_O_2_ for 25 min. Then we blocked the antigen using goat serum (Servicebio, G5001) for 30 min. We used MYD88 (1:20, CST, 4283S),CD68 (1:200, Servicebio, GB14043) and CD163 (1:600, Servicebio, GB13340) antibody overnight at 4°C to stain the slides, among which the two adjacent slides were stained with CD68 and CD163 separately. Then the slides were incubated with secondary antibodies (1:200, Servicebio, GB23303) for MYD88 and CD163 or secondary antibodies (1:200, Servicebio, GB23301) for CD68 50 min at room temperature. After adding DAB (DAKO, K5007) and hematoxylin (Servicebio, G1004) staining, slides were covered and observed by microscope (Grundium OCUS).

### Evaluation of the Immune Reactive Score (IRS)

We used the immune-reactive score (IRS) to evaluate the expression of MYD88. The IRS included the quantity of stained cells and intensity of immune staining. The percentage of stained cells in the positive cells was applied to define the reaction as negative (0%), 1+ (<10%), 2+ (10%–50%), 3+ (51%–80%), and 4+ (>80%). The intensity of staining was classified as: absent (0), weak (1+), moderate (2+), strong (3+). The final value of the analysis of each slide was then recorded through the obtained score by multiplying the two scores. Five scanning fields (400× magnification) were randomly chosen and evaluated independently by two pathologists using Grundium OCUS microscope. The average of the scores of each slide was figured out. The IRS score of MYD88 was divided into groups based on IDH1 mutation status or WHO grades and compared separately.

### Evaluation of Estimate, Immune, and Stromal Score

The ESTIMATE computational method in the “estimate” package in R software was applied to calculate the “estimate score,” “immune score,” and “stromal score” in gliomas (28).

### Identification of Differentially Expressed Genes and Functional Enrichment

We used the R package “limma” to figure out differentially expressed genes (DEGs) in the immune-score group and stromal-score group in glioma tissues. R language with package “pheatmap” was applied to produce the heatmap. And R package “clusterProfiler” was applied to conduct functional annotations, which include three types of GO (biological processes (BP), molecular functions (MF), and cellular components (CC)) and KEGG enrichment analysis. Terms with both p-value and q-value of <0.05 were considered significantly enriched.

### PPI Network and Cox Analysis for Screening MYD88 Gene

The PPI network was constructed using the Search Tool for the Retrieval of Interacting Genes/Proteins (STRING) database and rebuilt by the Cytoscape (Version 3.8.1). We used the nodes with confidence of interactive relationship larger than 0.99 to construct the network. The top 30 genes ranked by the connection edges were displayed in the barplot. The top 33 genes in univariate Cox analysis were depicted in the plot.

### Gene Set Enrichment Analysis

Gene set enrichment analysis (GSEA) was performed by the GSEA-4.1.0 using the Hallmark and C7 gene sets v7.2 downloaded from the Molecular Signatures Database (MSigDB) as the target sets. The whole transcriptome of all tumor samples was used for GSEA.

### Characteristics of the TME in Gliomas

We applied CIBERSORT to compute cell components of the tissues. Twenty-two categories of TIICs (such as plasma cells, natural killer cells, among others) were identified and calculated the relative proportions using CIBERSORT in R and the LM22 signature matrix. Correlation analysis between different TIIC subpopulations was achieved by the “corrplot” package. The “vioplot” package was applied to visualize the TIICs between MYD88 high expression and low expression group. The association between the expression of MYD88 and the TIICs was acquired using “limma,” “ggplot2,” “ggpubr,” and “ggExtra” packages.

### Statistical Analysis

The univariate Cox, survival, TME, gene difference, and clinical characteristics analyses were carried out in R (v.4.0.2). Wilcoxon signed-rank test was applied to compare gene expression differences and immune-reactive score (IRS). Kaplan-Meier analysis and log-rank test were used to conduct survival analysis. We used the “ggpubr” and “limma” packages to compute correlations between the expression of MYD88 and immune cells. Spearman’s or Pearson’s correlation test was conducted to evaluate the correlation of two variables. A p < 0.05 was considered to be statistically significant.

## Results

### Analysis Process

The analysis process is schematically shown in **Figure S1**. We downloaded transcriptome RNA-seq data of 703 cases from TCGA database. The 28 glioma patients without clinical data were not included in the survival analysis. The clinicopathological information of the remaining 670 glioma cases were displayed in **Table S1**. CIBERSORT and ESTIMATE algorithms were applied to calculate the composition of TIICs and the amount of immune and stromal component in gliomas separately. The DEGs contributing to the immune score and stromal score were figured out to construct PPI network and conduct univariate Cox regression analysis. Then we performed the intersection analysis between the top 30 core nodes in PPI network and top 33 significant factors in Cox regression analysis. And the MYD88 gene was found as the core gene in the analysis. Furthermore, we conducted the survival analysis, clinicopathological characteristics correlation analysis, Cox regression, GSEA, and correlation with TIICs.

### TME Scores Are Correlated With the Survival, Age, Gender, and WHO Grade of Gliomas

**Figure 1:**
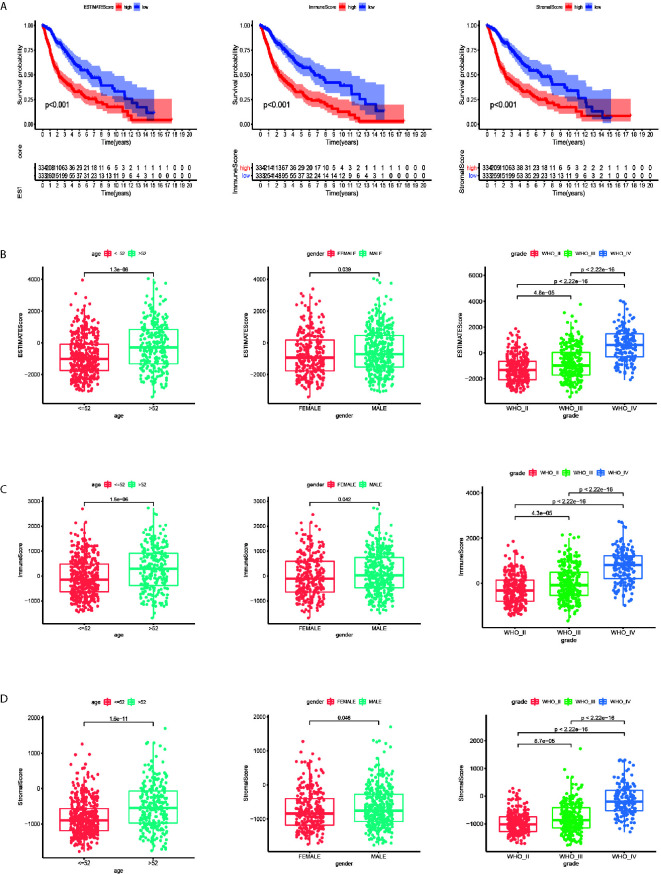
Tumor microenvironment scores are correlated with the survival, age, gender and WHO grade of gliomas. **(A)** The patients with higher ESTIMATE Score, Immune Score and Stromal Score (p < 0.001) tended to have longer overall survival time. **(B)** The ESTIMATE Scores were significantly higher in male, age > 52 years and higher WHO grade patients. **(C)** The Immune Scores were significantly higher in male, age > 52 years and higher WHO grade patients. **(D)** Male, age > 52 years and higher WHO grade patients tended to have higher Stromal Scores.

Kaplan–Meier analysis was applied to calculate the association between high and low TME scores divided by the median score. The higher score represented the larger proportion of the immune or stromal components. The patients with higher ESTIMATE Score (p < 0.001), Immune Score (p < 0.001) and Stromal Score (p < 0.001) tended to have longer survival time (**Figure 1A**). The ESTIMATE Scores were significantly higher in male patients (**Figure 1B**, p = 0.039), age > 52 years (**Figure 1B**, p < 0.001) and higher WHO grade (**Figure 1B**, p < 0.001). The Immune Scores were significantly higher in male patients (**Figure 1C**, p = 0.042), age > 52 years (**Figure 1C**, p < 0.001) and higher WHO grade (**Figure 1C**, p < 0.001). Male (**Figure 1D**, p = 0.046), age > 52 years (**Figure 1D**, p < 0.001), and higher WHO grade patients (**Figure 1D**, p < 0.001) had higher stromal scores.

### DEGs Obtained by the intersection of Immune Score and Stromal Score Showed Immune-Related Pathway Enrichment

**Figure 2:**
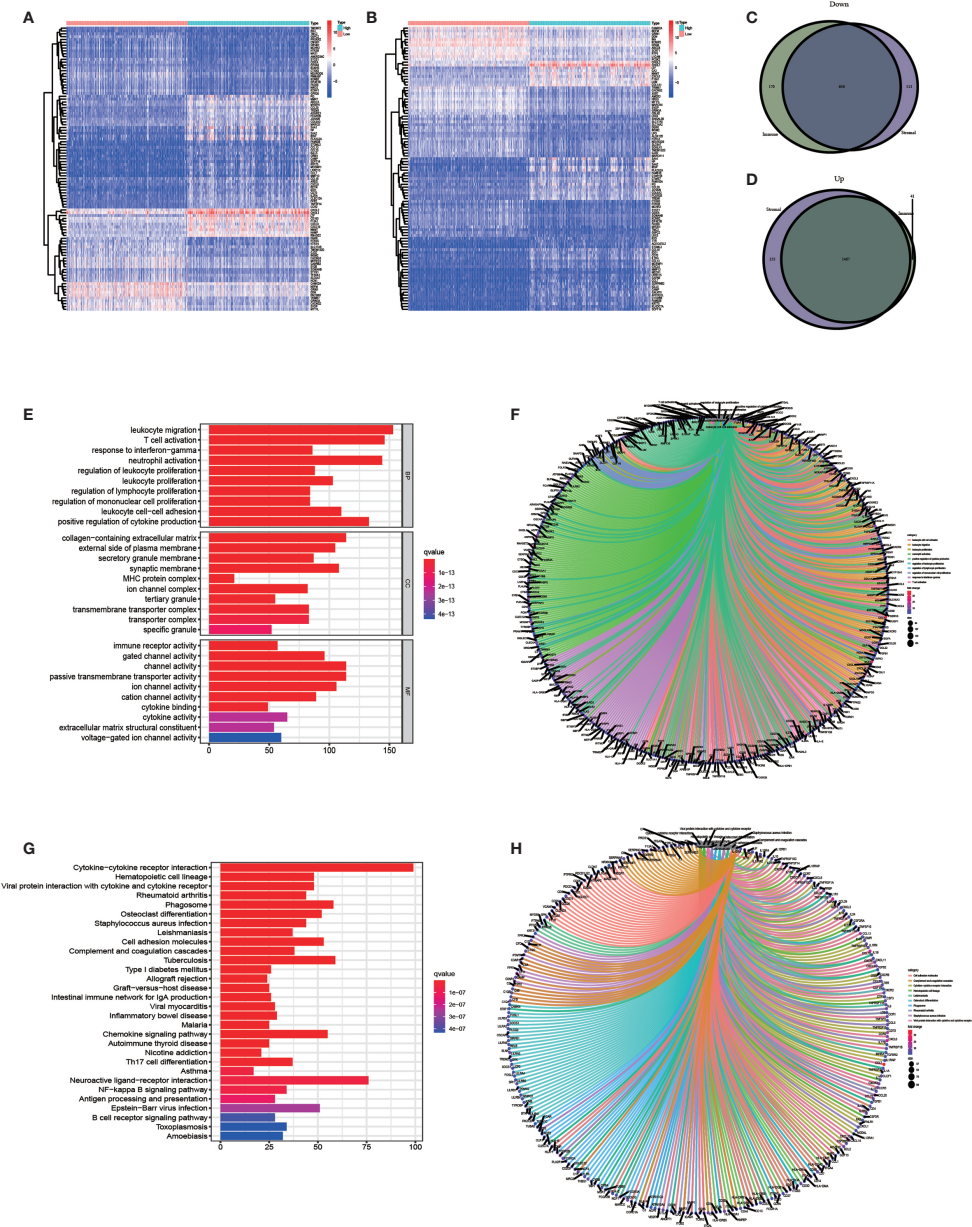
DEGs obtained by the intersection of Immune Score and Stromal Score showed Immune-related pathway Enrichment. **(A)** The heatmap of the DEGs of Immune Score difference. **(B)** The heatmap of the DEGs of Stromal Score difference. **(C)** A total of 636 genes were down regulated both in immune and stromal components. **(D)** A total of 1467 genes were up regulated both in immune and stromal components. **(E, F)** The GO analysis showed that the DEGs were mainly enriched in immune-related pathways. **(G, H)** The KEGG analysis showed that the DEGs were mainly enriched in immune-related pathways.

The comparison between high and low-score samples was conducted to figure out the gene profile alteration characteristics with regard to immune and stromal components (**Figures 2A, B**). A total of 806 genes were down regulated and 1508 genes were up regulated in the immune components (**Figures 2C, D**). There were 749 genes down regulated and 1822 genes up regulated in the stromal components (**Figures 2C, D** **)**. The Venn plot by the combination analysis showed 636 genes down regulated and 1467 genes up regulated both in immune and stromal components (**Figures 2C, D** **)**. The GO and KEGG pathway enrichment analyses were conducted based on the 2103 genes shared by the immune and stromal parts. We found that the DEGs were enriched in immune-related pathways, including leukocyte cell-cell adhesion, leukocyte migration, leukocyte proliferation, neutrophil activation, positive regulation of cytokine production, regulation of leukocyte proliferation, regulation of mononuclear cell proliferation, response to interferon-gamma, and T cell activation in GO analysis (**Figures 2E, F** **)**. The KEEG analysis also displayed the enrichment of cell adhesion molecules, complement and coagulation cascades, cytokine-cytokine receptor interaction, hematopoietic cell lineage, leishmaniasis, osteoclast differentiation, phagosome, rheumatoid arthritis, *Staphylococcus aureus* infection, and viral protein interaction with cytokine and cytokine receptor (**Figures 2G, H**). The functions of DEGs seemed to mainly map on immune-related activities.

### MYD88 Was Screened by the Intersection of PPI Network and Univariate Cox Analysis

**Figure 3:**
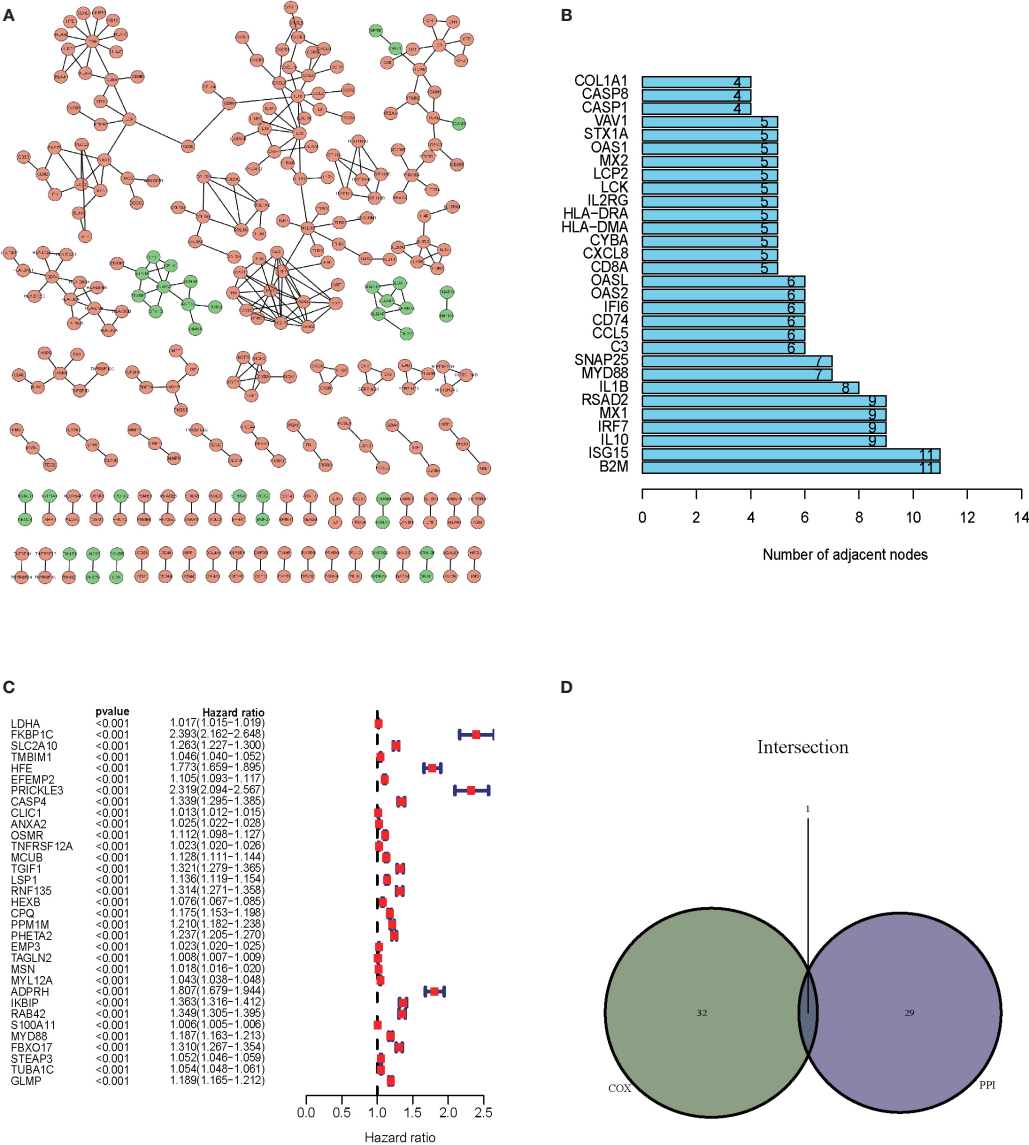
MYD88 was screened by the intersection of PPI network and univariate Cox analysis. **(A)** PPI network of the DEGs. **(B)** The top 30 proteins that had the maximum number of nodes in the PPI network. **(C)** Univariate Cox regression analysis for the survival of glioma patients. **(D)** The intersection analysis between the top 30 nodes in PPI network and the leading 33 genes ranked by the p-value of univariate Cox regression showed that MYD88 was the key gene.

PPI network (**Figure 3A**) was constructed from the STRING database using Cytoscape software (National Institute of General Medical Sciences [NIGMS] USA). There were 299 nodes and 311 edges based on the PPI network analysis (minimum required interaction score > 0.99, **Figure 3A** **)**. **Figure 3B** displayed the top 30 proteins that had the maximum number of nodes in the PPI network. Univariate Cox regression analysis for the survival of glioma patients was conducted to figure out the factors in 2103 DEGs (**Figure 3C**). Furthermore, we conducted the intersection analysis between the top 30 nodes in PPI network and the leading 33 genes ranked by the p-value of univariate Cox regression and found that MYD88 was the only gene in the analysis (**Figure 3D**).

### MYD88 Was Associated With the Overall Survival, Age, WHO Grade and Enriched in Immune Pathways

**Figure 4:**
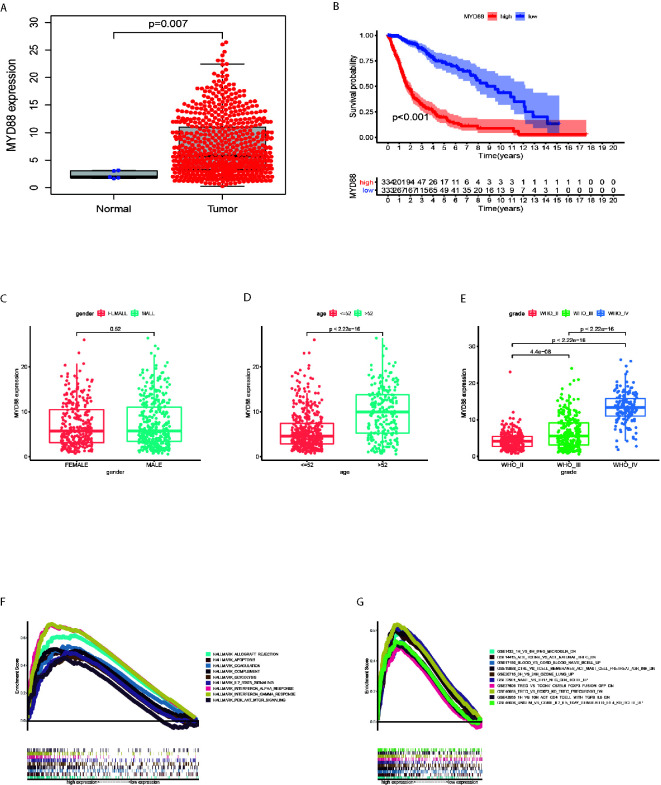
MYD88 was associated with the overall survival, age, WHO grade and enriched in immune pathways. **(A)** The MYD88 expression were significantly lower in normal tissues as compared to the glioma tissues. **(B)** MYD88 lower expression was associated with significantly longer overall survival. **(C)** The MYD88 expression was similar between male and female patients. **(D)** MYD88 expression was higher in patients with age > 52 years. **(E)** MYD88 expression was higher in patients with higher WHO grade. **(F)** The genes in MYD88 high-expression group were mainly enriched in immune related pathways. **(G)** For C7 collection defined by MSigDB, the immunologic gene sets, multiple immune functional gene sets were enriched in the high MYD88 expression group.

The MYD88 expression were significantly lower in normal tissues as compared to the glioma tissues (p = 0.007, **Figure 4A**). In glioma patients, MYD88 lower expression was associated with significantly longer overall survival (p < 0.001, **Figure 4B** **)**. MYD88 expression was higher in patients with age > 52 years (**Figure 4D**, p < 0.001) and higher WHO grade (**Figure 4E**, p < 0.001). MYD88 expression showed similar levels between male and female patients. (**Figure 4C**, p = 0.52) The genes in MYD88 high-expression group were mainly enriched in allograft rejection, apoptosis, coagulation, complement, glycolysis, IL2_STAT5_signaling, Interferon_Alpha_response, Interferon_Gamma_response, and PI3K_AKT_MTOR_signaling (**Figure 4F**). The immunologic gene sets, multiple immune functional gene sets, were enriched in the high MYD88 expression group in C7 collection (**Figure 4G** **)**.

### Macrophage M2 Accounted the Largest Portion of the TIICs

**Figure 5:**
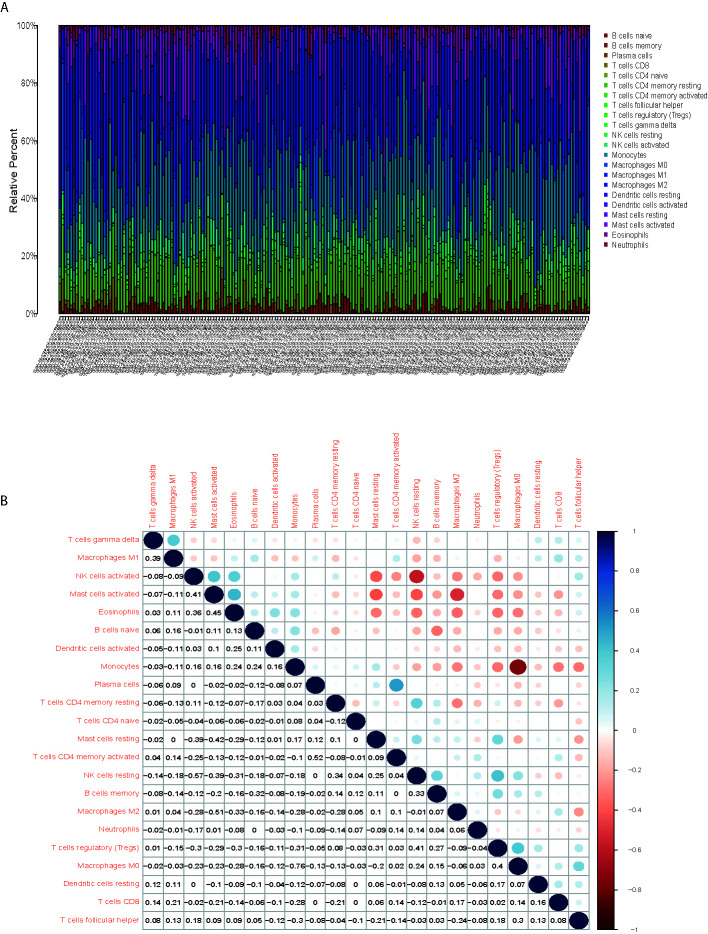
Macrophage M2 accounted the largest portion of the TIICs. **(A)** The first three largest portion of immune cells were Macrophage M2, Monocytes and CD4 memory resting cells. **(B)** correlation of different immune cells showed that the Macrophage M2 and Mast cells activated was negatively correlated.

The CIBERSORT was applied to calculate the proportions of twenty-two immune cell types in the each sample (**Figure 5A**). The first three largest portion of immune cells were Macrophage M2 (27.6%, **Figure 5A**), Monocytes (19.0%, **Figure 5A**) and CD4 memory resting cells (15.6%, **Figure 5A**). The correlation of different immune cells was displayed in **Figure 5B**, which showed that Macrophage M2 and Mast cells activated (r = −0.51) was negatively correlated.

### MYD88 Was Associated With the TIICs of TME in Glioma Patients

**Figure 6:**
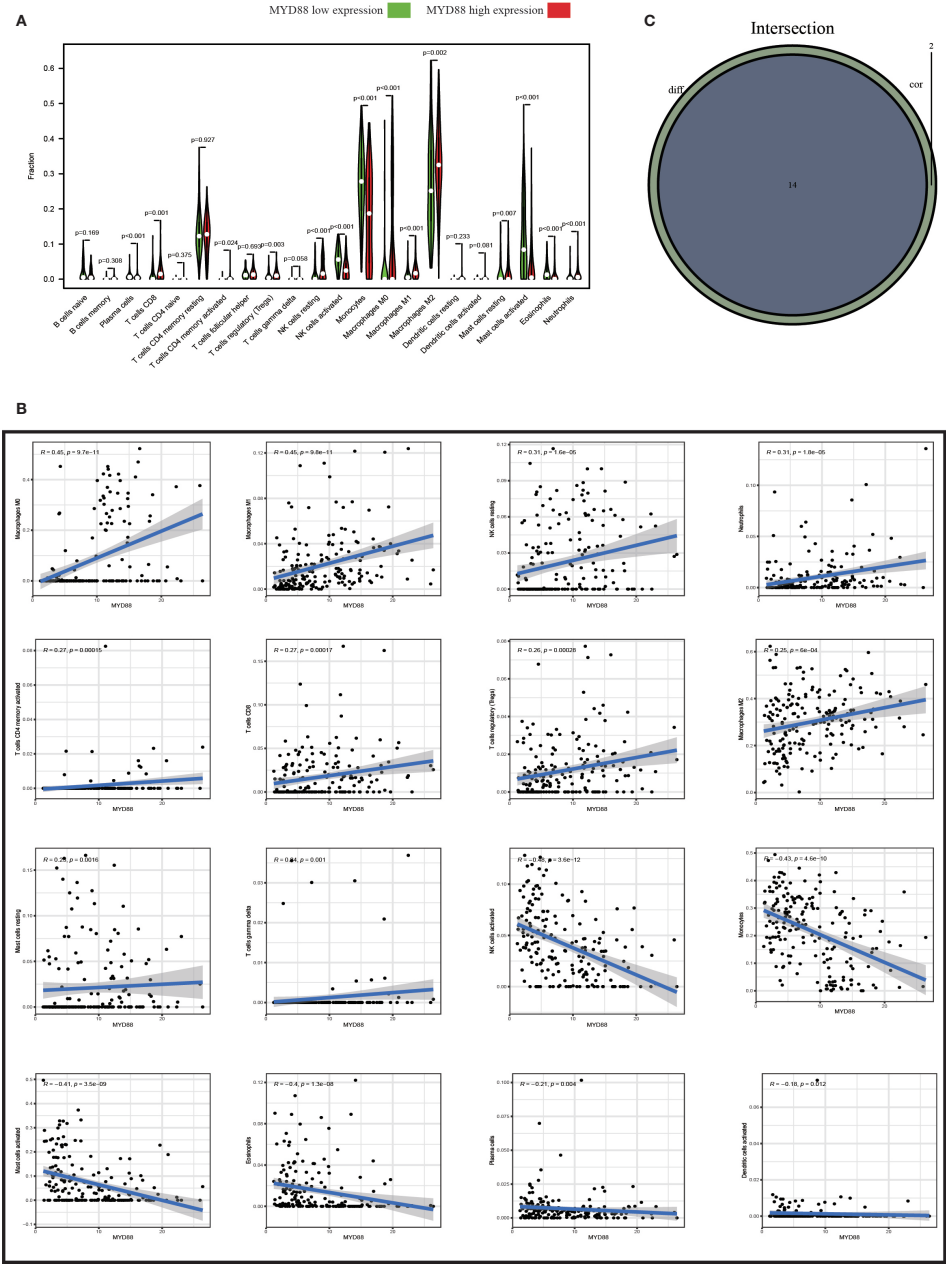
MYD88 was associated with the TIICs of TME in glioma patients. **(A)** The Macrophage M0, Macrophage M1 and Macrophage M2 were significantly up-regulated in MYD88 high-expression group. However, the Monocytes, Mast cells activated and Eosinophils were significantly up-regulated in MYD88 low-expression group. **(B)** The MYD88 expression was positively associated with Macrophage M0, Macrophage M1, and Macrophage M2. And MYD88 was negatively correlated with NK cell activated, Monocytes and Mast cells activated. **(C)** Intersection analysis showed that 14 TIICs were significantly associated with MYD88 expressions using both difference and correlation analysis.

To explore the impact of MYD88 on the TME of glioma patients, the patients were divided into high and low-expression groups. We found that the macrophage M0 (p < 0.001, **Figure 6A** **),** macrophage M1 (p < 0.001, **Figure 6A** **)** and macrophage M2 (p = 0.002, **Figure 6A**) were significantly up-regulated in MYD88 high-expression group. However, the monocytes (p < 0.001, **Figure 6A**), mast cells activated (p < 0.001, **Figure 6A**), and eosinophils (p < 0.001, **Figure 6A** **)** were significantly up-regulated in MYD88 low-expression group.

The correlations of MYD88 expression with TIICs were also calculated. We found that the MYD88 expression was positively associated with macrophage M0 (r = 0.45, p < 0.001, **Figure 6B**), macrophage M1 (r = 0.45, p < 0.001, **Figure 6B** **)**, and macrophage M2 (r = 0.25, p < 0.001, **Figure 6B**). And MYD88 was negatively correlated with NK cell activated (r = −0.48, p < 0.001, **Figure 6B**), monocytes (r = −0.43, p < 0.001, **Figure 6B**) and mast cells activated (r = −0.41, p < 0.001, **Figure 6B**). Intersection analysis showed that 14 TIICs were significantly associated with MYD88 expressions using both difference and correlation analysis (**Figure 6C**).

### MYD88 Expression Was Associated With the IDH Mutant Status, Age, and WHO Grade Both in CGGA Database and Clinical Cases

We validated the above findings both in CGGA database and clinical cases in our hospital. In CGGA, we found that MYD88 expression increased with the WHO grade (p < 0.001, **Figure S2A**). IDH 1 mutant status was associated with lower MYD88 expression (p < 0.001, **Figure S2B**). MYD88 expression mainly manifested significantly lower in WHO III (p < 0.001, **Figure S2C**) and WHO IV (p < 0.001, **Figure S2C**) grade IDH 1 mutant patients. Moreover, the MYD88 gene methylation decreased significantly with the WHO grade (p < 0.001, **Figure S3**).

**Figure 7:**
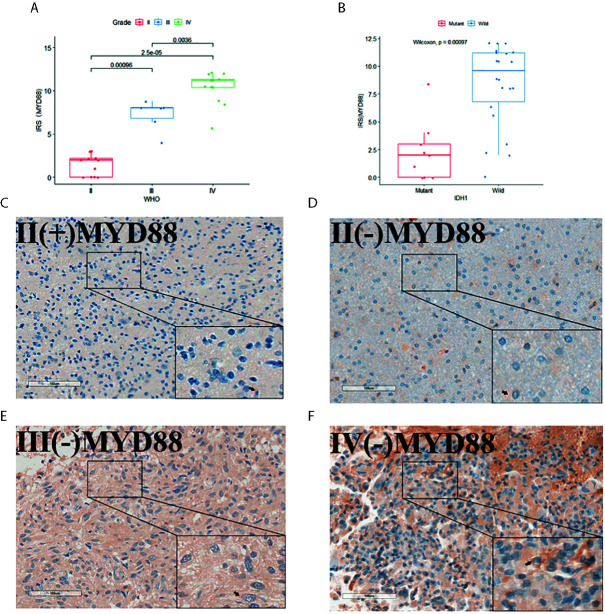
The MYD88 IHC staining of the 31 glioma patients. **(A)** MYD88 expression was significantly higher in high-grade gliomas. **(B)** In the IDH1 mutant cases, the MYD88 was significantly lower as compared to the cases with IDH1 wild cases. **(C–F)** IHC image of example glioma patients with WHO grade II/IDH1(+), WHO grade II/IDH1 (–), WHO grade III/IDH1 (–) and WHO grade IV/IDH1 (–) pathology results separately.

**Figure 8:**
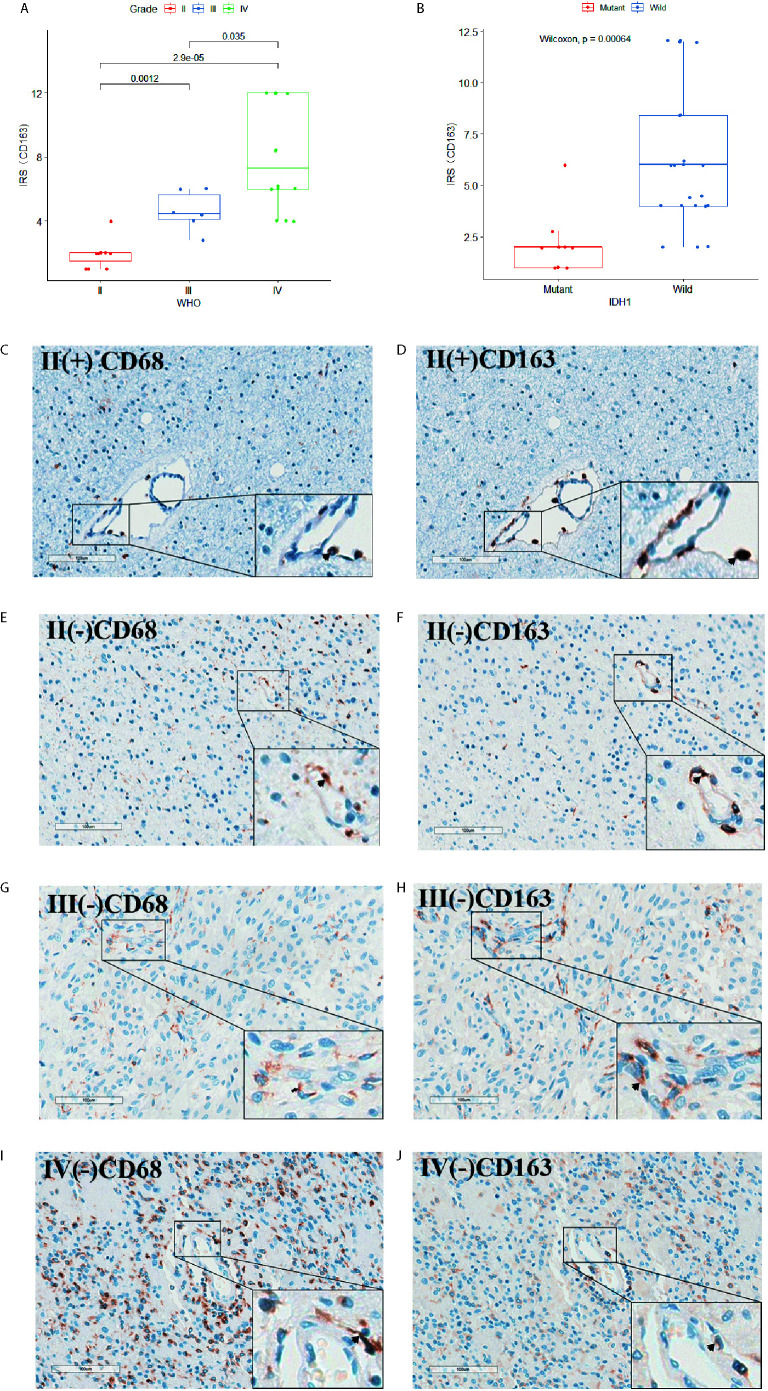
The CD68 and CD163 IHC staining of the 31 glioma patients. **(A)** CD163 expression was significantly higher in high-grade gliomas. **(B)** In the IDH1 mutant cases, the CD163 was significantly lower as compared to the cases with IDH1 wild cases. **(C–J)** CD68 and CD163 IHC staining image of adjacent slides in clinical glioma patients with WHO grade II/IDH1(+), WHO grade II/IDH1 (–), WHO grade III/IDH1 (–) and WHO grade IV/IDH1 (–) pathology results separately.

Thirty-one IHC staining plates were photographed and analyzed using IRS. The basic clinicopathological information was listed in **Table 1**. We found that the MYD88 expression was significantly higher in high-grade gliomas (p < 0.001 (WHO II vs WHO III), p = 0.0036 (WHO III vs WHO IV), **Figures 7A, D–F**). The M2 macrophage marker CD163 was expressed higher in high-grade gliomas (p = 0.0012 (WHO II vs WHO III), p = 0.035 (WHO III vs WHO IV), **Figures 8A, F–H**). CD68 was used as the tumor associated macrophage marker (**Figures 8C, E, G, I**). And in the IDH1 mutant cases, the MYD88 was significantly lower as compared to the cases with IDH1 wild cases (p < 0.001, **Figures 7B–F**). The CD163 was expressed lower in IDH1 mutant cases (p < 0.001, **Figures 8B, D, F, H, J**).

**Table 1:** Clinicopathological information of the glioma patients.

Patients	Gender	Age (years)	Histology	WHO grade	IDH1	IRS (MYD88)	IRS (CD163)
P1	M	47	Astrocytoma	Ii	Mutant	0	1
P2	M	37	Oligodendroglioma	Ii	Mutant	2.2	1
P3	M	44	Oligodendroglioma	Ii	Mutant	0	2
P4	F	61	Oligodendroglioma	Ii	Mutant	1	2
P5	M	54	Astrocytoma	Ii	Mutant	2	1
P6	M	35	Oligodendroglioma	Ii	Mutant	0	2
P7	F	33	Astrocytoma	Ii	Mutant	3	2
P8	F	58	Astrocytoma	Ii	Wild	2.2	2
P9	M	34	Astrocytoma	Ii	Wild	3	2
P10	F	12	Oligodendroglioma	Ii	Wild	0	2
P11	M	32	Astrocytoma	Ii	Wild	2	4
P12	F	27	Anaplastic oligodendroglioma	Iii	Mutant	4	2.8
P13	M	27	Anaplastic astrocytoma	Iii	Wild	8	4.5
P14	F	50	Anaplastic astrocytoma	Iii	Wild	8	4.4
P15	M	29	Anaplastic astrocytoma	Iii	Wild	6.4	4
P16	F	68	Anaplastic astrocytoma	Iii	Wild	8	6
P17	F	75	Anaplastic astrocytoma	Iii	Wild	8.8	6
P18	M	41	Glioblastoma	Iv	Mutant	8.4	6
P19	M	39	Glioblastoma	Iv	Wild	11.2	8.4
P20	M	64	Glioblastoma	Iv	Wild	12	4
P21	M	42	Glioblastoma	Iv	Wild	12	8.4
P22	M	47	Glioblastoma	Iv	Wild	11.2	12
P23	F	56	Glioblastoma	Iv	Wild	12	6
P24	M	34	Glioblastoma	Iv	Wild	11.2	12
P25	F	34	Glioblastoma	Iv	Wild	11.2	6
P26	M	43	Glioblastoma	Iv	Wild	10.4	6.2
P27	F	61	Glioblastoma	Iv	Wild	10.4	12
P28	M	69	Glioblastoma	Iv	Wild	8.8	4
P29	F	49	Glioblastoma	Iv	Wild	5.6	4
P30	M	65	Glioblastoma	Iv	Wild	11.4	12
P31	M	49	Glioblastoma	Iv	Wild	10.4	12

IRS means average immune reactive score based on five random fields in each case.

## Discussion

This study was conducted to identify the TME-related gene associated with the survival and the WHO grade in glioma patients based on the TCGA database. The results were also testified in CGGA database and clinical glioma tissues in our hospital. We found that the TME was associated with the survival of glioma patients. The differentially expressed genes were mainly enriched in immune pathways. MYD88 was obtained by the intersection of PPI analysis and univariate Cox analysis. Further analysis revealed that MYD88 was over expressed in gliomas and associated with the survival, WHO grade and TIICs especially the macrophage M2. The similar results were acquired in CGGA database and clinical cases. Besides that, MYD88 was also down regulated in the IDH1 mutant gliomas. MYD88 gene methylation was lower in higher grade gliomas.

### TME Characteristics of Glioma

The TME plays a pivotal role in solid tumors through biochemical and biophysical factors generated by cancer reprogramming of cell-cell and cell-ECM interactions (29). Recent advances indicated that tumor TME played a complex role in the tumor development and metastasis (30). It is of great significance to explore the TME profile of gliomas. This article used the ESTIMATE to calculate the immune score and stromal score, which could be used to reflect the purity of the tumor. We observed that patients with high immune scores or stromal scores had a shorter overall survival as compared with those with low scores. Then 2103 DEGs between patients with high scores and those with low scores were figured out. GO and KEGG pathway enrichment analysis revealed that the DEGs mainly participated in the immune pathways, such as leukocyte cell-cell adhesion, leukocyte migration, leukocyte proliferation, neutrophil activation, positive regulation of cytokine production, regulation of leukocyte proliferation, regulation of mononuclear cell proliferation, response to interferon-gamma and T cell activation, the enrichment of cell adhesion molecules, complement and coagulation cascades, cytokine-cytokine receptor interaction, hematopoietic cell lineage, leishmaniasis, osteoclast differentiation, phagosome, rheumatoid arthritis, staphylococcus aureus infection, and viral protein interaction with cytokine and cytokine receptor. These results were in accordance with several studies. Xiangyang Deng et al. conducted the IRG expression and immune infiltration pattern in TME of lower-grade glioma (WHO grade II/III) (16). They found that grade III or IDH1 wild type gliomas had both higher immune and stromal scores (31).They also found that ESTIMATE algorithms‐based scores were meaningful in subtype classification of glioblastomas and affected prognosis. Li Yong et al. reported that increased immune and stromal scores were closely related with advanced glioma grade and poor prognosis (32). And the GO and KEGG analyses revealed that the majority of the DEGs were involved in immunologic process. These results indicated that the TME was characterized by immune cells reorganization and dysregulation, which significantly influenced the prognosis and tumor progression of glioma patients.

### MYD88 Might Participate in the Vicious Circle of Tumor Cells Progression and M2 Macrophage Polarization

TME immune cells regulate tumor cells through cytokines and chemokines (33, 34). It is of great importance to identify the prognostic risk factors associated with TME immune cells. By the intersection of top 30 PPI network core genes and top 33 core genes of univariate Cox analysis, we figured out only one core gene MYD88. The MYD88 expression was significantly lower in normal tissues and high expression was associated with shorter survival time, older age, and higher WHO grade. GSEA analyses showed that MYD88 expression mainly enriched in immune pathways. This study firstly figured out the MYD88 gene as the key gene related with TME immunity. MYD88 protein is a commonly expressed adaptor protein in the cytoplasm (35). It plays a key role in the toll-like receptor (TLR) and interleukin-1 receptor (IL-1R) signaling pathways responding to the pathogen-associated molecular patterns (PAMPs) produced by infectious microbes and damage-associated molecular pattern molecules (DAMPs) derived from injured host cells (18). TLRs belong to type I transmembrane proteins, including an N-terminal, leucine-rich repeat domain, a single transmembrane link, and a C-terminal cytoplasmic domain (36). They are expressed by immune cells (including monocytes/macrophages, neutrophils, myeloid dendritic cells, mast cells, B lymphocytes, among others) that participate in the innate immune system. MYD88 protein includes three domains: an N-terminal death domain (DD), a short intermediate domain (ID), and a C-terminal Toll/IL-1R (TIR) domain (37). When an endogenous-deprived ligand or exogenous stimuli interacts with the extracellular domain of TLRs/IL-1R receptors (except for TLR3), the TIR domain forms a dimer to recruit MYD88 proteins. Binding to TIR domains, MYD88 activates transcription factors, including nuclear factor-kappa B (NF-κB), activator protein-1 (AP-1), and interferon regulatory factors (IRFs) through the interaction with the DD domains of IRAK2/4 (38). Previous study revealed that MYD88 might promote tumor cell survival through IRAK-mediated NF-κB signaling in colorectal carcinoma cells (39). Furthermore, it may also contribute to damage DNA self-repairing by RAS-extracellular signal-regulated kinase (ERK) pathway in colon cancer cells (40). Jinyue Hu et al. found that the activation of TLR4 reverses tumor differentiation in human glioma U251 cells *via* Notch pathway, which was MYD88-dependent (22).

M2 macrophages, monocytes, and CD4 memory resting cells were the largest three kinds of the TIICs. Moreover, the MYD88 expression was positively associated with macrophage M2 and negatively associated with the monocytes. We also found that the M2 markers CD68 and CD163 were highly expressed in high-grade gliomas. Xiangyang Deng et al. applied unsupervised cluster analysis and identified that CD8+ T cells and macrophages were significantly associated with LGG outcomes (16).

There are mainly two activated forms of macrophages. Macrophages M1 phenotype have the function of anti-tumor immunity, proinflammatory activity, and the induction of T-cell responses (41). Macrophages M2 phenotype play an important role in tumor proliferation, invasion, metastasis, and neo‐angiogenesis (42, 43). Moreover, macrophages M2 phenotype can also compromise the efficacy of anticancer drugs. Their abundance in tumors is, therefore, associated with the survival of the patients (44, 45). Aurobind Vidyarthi et al. found that M2 macrophages were significantly higher in high-grade gliomas and could lead to the systemic and local immune suppression, which could compromise the treatment of immunotherapy (46). Katyayni Vinnakota et al. reported that the activation of TLRs converts microglia into a glioma-supportive phenotype through upregulation of membrane type 1 matrix metalloprotease (MT1-MMP), depending on signaling *via* the TLR adaptor molecule MYD88 (19). Qi Yuan et al. reported that MYD88 signaling in activated myofibroblasts in colitis-associated cancer mouse model increases the secretion of osteopontin (OPN) to promote M2 polarization *via* binding to α_v_β_3_ and CD44 and activating the STAT3/PPARγ pathway (47).

Based on previous findings, we could deduce that MYD88 and TLRs/IL-1R pathway might both participate in the cancer cell progression and the polarization of M2 macrophage. And the interaction between them might make the TME worse facilitating the invasion, metastasis, and proliferation of the tumor. However, because of the limited studies, the function and mechanism of MYD88 in the polarization of M2 and the interaction with glioma cells still need to be further explored.

### Validation in CGGA Database and Clinical Patients

We validated the above findings both in CGGA database and Thirty-one clinical cases in our hospital. In CGGA, we found that MYD88 expression increased with the WHO grade. IDH1 mutant status was associated with lower MYD88 expression. MYD88 expression mainly manifested significantly lower in WHO III and WHO IV grade IDH1 mutant patients compared with the IDH1 wild type group separately. Moreover, the MYD88 gene methylation decreased significantly with the WHO grade. Thirty-one IHC staining plates were photographed and analyzed using IRS. We found that the MYD88 expression was significantly higher in high-grade gliomas. And in the WHO IV grade tissues, the MYD88 was significantly lower in IDH1 mutant cases. IDH1 and methylation of O (6)-methylguanine DNA methyltransferase (MGMT) promoter are important biomarkers for GBM patients. The IDH1 mutation is common in lower grade gliomas. And 12% of GBM patients have IDH1 mutation (48, 49). The IDH1 mutation is associated with increased survival time of the patients (50). Xiangyang Deng et al. found that IDH1 wild type gliomas had both higher immune and stromal scores (16). These indicated that IDH1 mutant might be related with the downregulation of MYD88 expression and less inflammatory responses in glioma TME, which could benefit the prognostics of glioma patients.

DNA methylation is an important regulator method of gene expression. In general, hyper-methylation of promoter regions decreases gene expression (25). For instance, MGMT plays a significant role in maintaining genomic integrity by repairing cell damage induced by chemotherapeutic agents (51). MGMT promoter methylation can decrease its expression and correlates with improved overall survival in GBM patients during the treatment of chemotherapy (52). Therefore, it contributes to chemotherapy sensitivity in GBM patients (53). Wen Wang et al. established an eight-gene signature (C9orf64, OSMR, MDK, MARVELD1, PTRF, MYD88, BIRC3, RPP25) to divide GBMs into two groups based on TCGA database. They reported that low risk group had a significantly higher MYD88 methylation (54). These evidences indicate that DNA methylation might participate in the regulation of MYD88 expression in gliomas.

To sum up, this article explored the TME alteration characteristics in gliomas and figured out a core gene MYD88, which played a significant role in the immune responses affecting the prognostics of glioma patients. The results were validated in CGGA database and clinical cases in our hospital.

## Limitations

This is only a preliminary study about the TME profiles and possible related genes based on the TCGA and CGGA databases. Further experiments need to be conducted to verify the mechanism of MYD88 in the development of TME of gliomas, which might provide a new target gene and pathway in the treatment as an optional therapy combined with traditional and immunotherapy methods.

## Conclusions

MYD88 gene played a pivotal role in the TME immune responses by exert influence on the overall survival and histology of glioma patients. Its related pathway in the M2 macrophage and glioma cell progression might serve as a potential target for future immunotherapy.

## Data Availability Statement

The original contributions presented in the study are included in the article/**Supplementary Material**. Further inquiries can be directed to the corresponding author.

## Ethics Statement

The studies involving human participants were reviewed and approved by the clinical research ethics committee of the Huashan Hospital. Written informed consent to participate in this study was provided by the participants’ legal guardian/next of kin.

## Author Contributions

QG and JZ designed the study. QG and XX conducted the statistical analysis and the experiment. QG and XX wrote the article. JZ revised the manuscript. All authors contributed to the article and approved the submitted version.

## Funding

This work was supported by Shanghai Municipal Science and Technology Major Project (2018SHZDZX03), ZHANGJIANG LAB and Join Breakthrough Project for New Frontier Technologies of the Shanghai Hospital Development Center (SHDC12016120).

## Conflict of Interest

The authors declare that the research was conducted in the absence of any commercial or financial relationships that could be construed as a potential conflict of interest.

## Supplementary Material

Supplementary Table 1 The clinicopathological characteristics of glioma patients from TCGA.

Supplementary Figure 1 The analysis procedures of this study.

Supplementary Figure 2 MYD88 expression was associated with the IDH mutant status, age and WHO grade in CGGA database. **(A)** MYD88 expression increased with the WHO grade. **(B)** IDH 1 mutant status was associated with lower MYD88 expression. **(C)** MYD88 expression mainly manifested significantly lower in WHO III and WHO IV grade IDH 1 mutant patients.

Supplementary Figure 3 The MYD88 gene methylation decreased significantly with the WHO grade.
