# Supplementary material for: MYD88 Is a Potential Prognostic Gene and Immune Signature of Tumor Microenvironment for Gliomas
Source: Front Oncol. 2021 Apr 7;11:654388. doi: 10.3389/fonc.2021.654388 (PMC8059377; doi:10.3389/fonc.2021.654388)
Supplement: Supplementary Table 1 — The clinicopathological characteristics of glioma patients from TCGA. [file Table_1.docx]

Table S1. Clinicopathological characteristics of glioma patients from TCGA

| Clinical characteristics |  | | Total  (670) | | % |
| --- | --- | --- | --- | --- | --- |
| Age at diagnosis (y)  Gender  Grade | age <=52 (median age*)  age >52 (median age)  Male  Female  Ⅱ  Ⅲ  Ⅳ | 422  248  386  284  248  261  161 | | 62.99%  37.01%  57.61%  42.39%  37.01%  38.96%  24.03% | |

*The median age was divided by the clinical data of 1114 cases in TCGA database
