# Supplementary figures and images for: MYD88 Is a Potential Prognostic Gene and Immune Signature of Tumor Microenvironment for Gliomas
Source: Front Oncol. 2021 Apr 7;11:654388. doi: 10.3389/fonc.2021.654388 (PMC8059377; doi:10.3389/fonc.2021.654388)

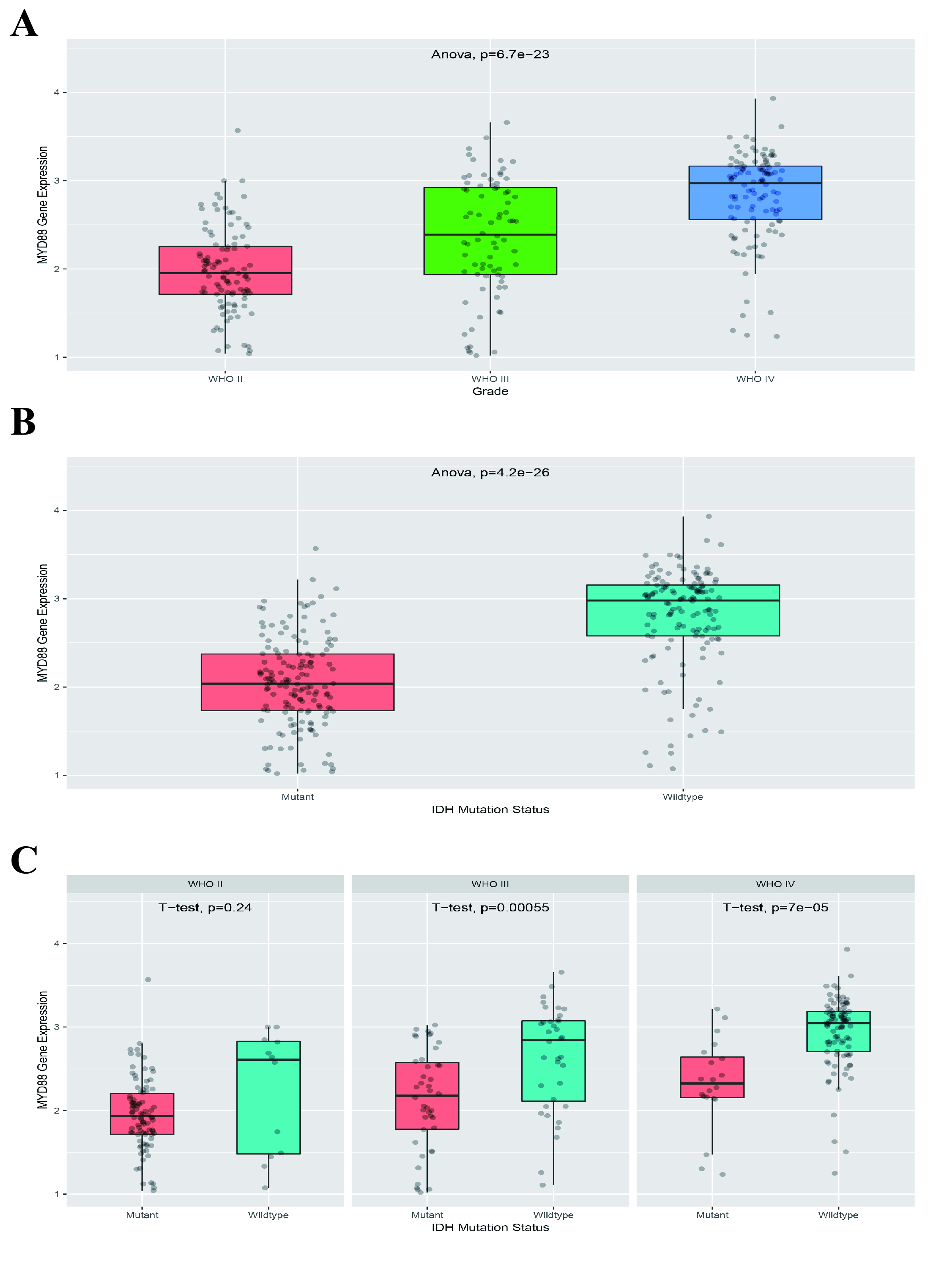

Supplement: Supplementary Figure 2 — MYD88 expression was associated with the IDH mutant status, age and WHO grade in CGGA database. (A) MYD88 expression increased with the WHO grade. (B) IDH 1 mutant status was associated with lower MYD88 expression. (C) MYD88 expression mainly manifested significantly lower in WHO III and WHO IV grade IDH 1 mutant patients. [file Image_2.jpeg]

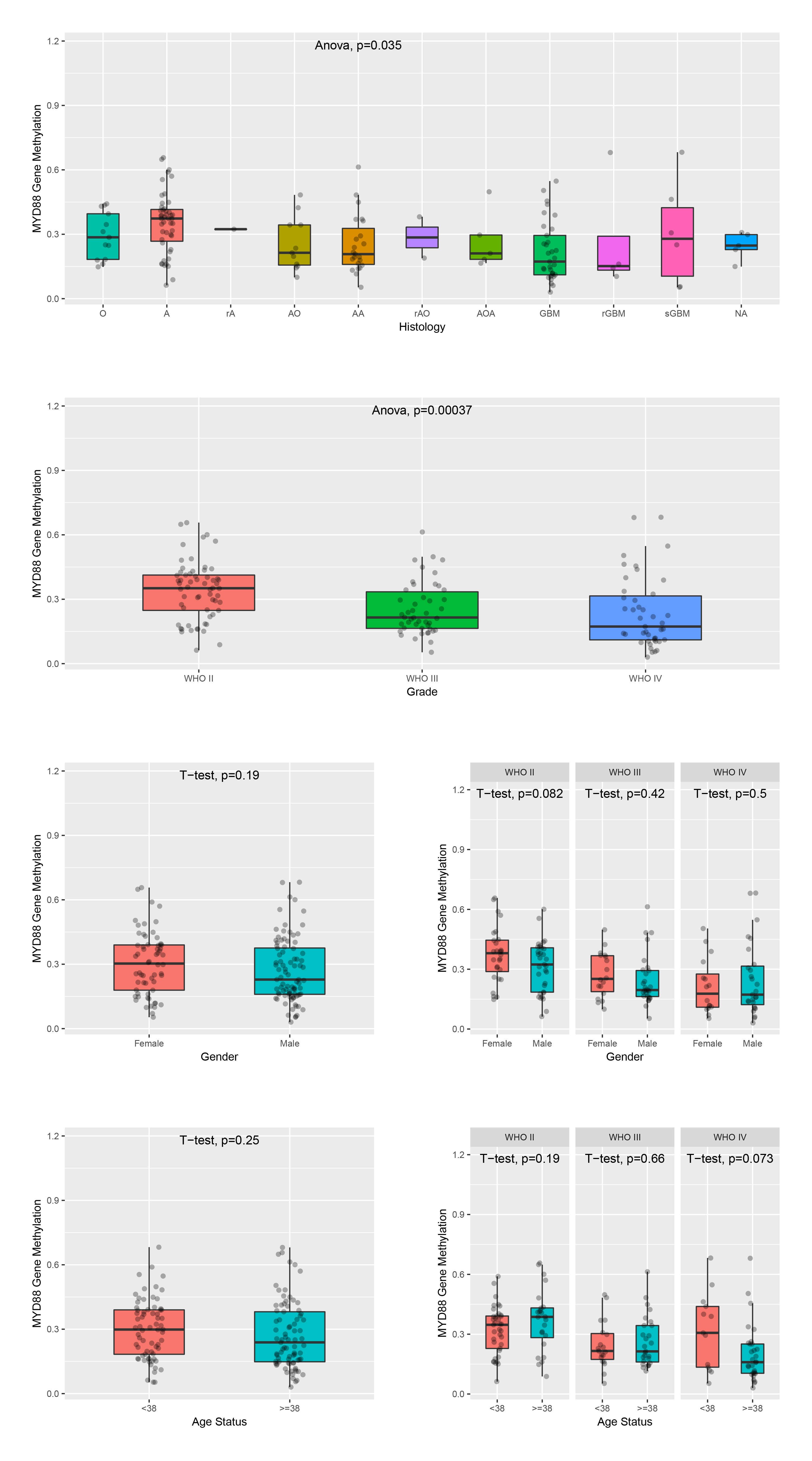

Supplement: Supplementary Figure 3 — The MYD88 gene methylation decreased significantly with the WHO grade. [file Image_3.jpeg]
